# The application of short and highly polymorphic microhaplotype loci in paternity testing and sibling testing of temperature-dependent degraded samples

**DOI:** 10.3389/fgene.2022.983811

**Published:** 2022-09-26

**Authors:** Dan Wen, Hao Xing, Ying Liu, Jienan Li, Weifeng Qu, Wei He, Chudong Wang, Ruyi Xu, Yi Liu, Hongtao Jia, Lagabaiyila Zha

**Affiliations:** ^1^ Department of Forensic Medicine, School of Basic Medical Sciences, Central South University, Changsha, China; ^2^ Xiangya Stomatological Collage, Central South University, Changsha, China

**Keywords:** forensic, microhaplotype, degraded samples, paternity testing, sibling testing

## Abstract

Paternity testing and sibling testing become more complex and difficult when samples degrade. But the commonly used genetic markers (STR and SNP) cannot completely solve this problem due to some disadvantages. The novel genetic marker microhaplotype proposed by Kidd’s research group combines the advantages of STR and SNP and is expected to become a promising genetic marker for kinship testing in degraded samples. Therefore, in this study, we intended to select an appropriate number of highly polymorphic SNP-based microhaplotype loci, detect them by the next-generation sequencing technology, analyze their ability to detect degraded samples, calculate their forensic parameters based on the collected 96 unrelated individuals, and evaluate their effectiveness in paternity testing and sibling testing by simulating kinship relationship pairs, which were also compared to 15 STR loci. Finally, a short and highly polymorphic microhaplotype panel was developed, containing 36 highly polymorphic SNP-based microhaplotype loci with lengths smaller than 100 bp and *A*_*e*_ greater than 3.00, of which 29 microhaplotype loci could not reject the Hardy-Weinberg equilibrium and linkage equilibrium after the Bonferroni correction. The CPD and CPE of these 29 microhaplotype loci were 1-2.96E-26 and 1-5.45E-09, respectively. No allele dropout was observed in degraded samples incubated with 100°C hot water for 40min and 60min. According to the simulated kinship analysis, the effectiveness at the threshold of 4/−4 reached 98.39% for relationship parent-child vs. unrelated individuals, and the effectiveness at the threshold of 2/−2 for relationship full-sibling vs. unrelated individuals was 93.01%, which was greater than that of 15 STR loci (86.75% for relationship parent-child vs. unrelated individuals and 81.73% for relationship full-sibling vs. unrelated individuals). After combining our 29 microhaplotype loci with other 50 short and highly polymorphic microhaplotype loci, the effectiveness values at the threshold of 2/−2 were 82.42% and 90.89% for relationship half-sibling vs. unrelated individuals and full-sibling vs. half-sibling. The short and highly polymorphic microhaplotype panel we developed may be very useful for paternity testing and full sibling testing in degraded samples, and in combination with short and highly polymorphic microhaplotype loci reported by other researchers, may be helpful to analyze more distant kinship relationships.

## 1 Introduction

Kinship testing is a major area of forensic research, and often includes paternity testing and sibling testing. Paternity testing refers to the identification of suspected relationships between parent and child, and sibling testing refers to the identification of suspected relationships between brothers and (or) sisters. Siblings are called full siblings if they are of the same father and mother, and half-siblings if they are of the half-blood. Paternity testing and sibling testing are mainly applied to disputed kinship testing, kinship testing in immigration and property inheritance, and kinship testing in major disasters and accidents (Wenk, 2004). Paternity testing can be easily solved by finding genetic exclusions. But siblings share only part of their genetic material, sibling testing is full of risk and uncertainty. Moreover, if the sample is degraded, it will make kinship testing more special and complicated.

At present, the most common method for paternity testing and sibling testing is based on the short tandem repeats (STR) typing technology, which has the advantages of high sensitivity, strong identification ability, high result accuracy, and comprehensive database (Butler, 2006). However, mismatch loci are frequently observed in relatives due to high mutation rates, and degraded samples cannot be conducive to analysis due to long amplicons (Lai and Sun, 2003). Some forensic genetics experts recommend the use of single nucleotide polymorphism (SNP) genetic markers to supplement kinship testing. SNP has obvious advantages, such as low mutation rate, large number, and short length. However, due to limited genetic information, a large number of SNPs are needed to find true genetic exclusion (Pakstis et al., 2007). Combining the advantages of STR and SNP, Kidd’s research group proposed a new genetic marker-microhaplotype, which has special advantages including lower mutation rate than STR, more polymorphic than SNP, short length, and no stutter peak. These microhaplotype loci can also be used for ancestry inference, personal identification, kinship testing, and mixture sample analysis (Kidd et al., 2014). So, short and highly polymorphic microhaplotype loci may be promising genetic markers for paternity testing and sibling testing in degraded samples.

Some studies have reported the use of microhaplotype loci in kinship testing (Zhu et al., 2019a; Zhu et al., 2019b; de la Puente et al., 2020; Kureshi et al., 2020; Qu et al., 2020; Sun et al., 2020; Staadig and Tillmar, 2021; Wen et al., 2021; Wu et al., 2021; Bai et al., 2022). In 2019, Zhu published two kinship testing studies, but a limited number of microhaplotype loci were reported, which may only be suitable for paternity testing (Zhu et al., 2019a; Zhu et al., 2019b). Then, to improve the effectiveness of kinship testing, Sun (Sun et al., 2020), Kureshi (Kureshi et al., 2020), Wen (Wen et al., 2021), and Wu (Wu et al., 2021) previously reported greatly highly polymorphic microhaplotype loci for kinship testing, which obtained good kinship detection ability. But most of the microhaplotype loci in these four studies were larger than 100 bp in length, for example, the mean length of 216 bp for Sun, 123 bp for Kureshi, 215 bp for Wen, and 164 bp for Wu, of which some microhaplotype loci may not be useful for kinship testing in severely degraded samples. Meanwhile, Staadig (Staadig and Tillmar, 2021), de la Puente (de la Puente et al., 2020), Qu (Qu et al., 2020), and Bai (Bai et al., 2022) published many short microhaplotype loci smaller than 100 bp in length, but the polymorphisms of some loci were limited. To obtain sufficient effectiveness of kinship testing, a large number of loci need to be detected, which may lead to multiplex amplification difficulties, linkage disequilibrium, as well as time-consuming and labor-intensive. Therefore, it is necessary to develop a novel panel containing an appropriate number of short and highly polymorphic microhaplotype loci for paternity testing and sibling testing in degraded samples.

In our previous studies, we screened many multi-allelic SNPs (Zha et al., 2012), some of which could form microhaplotype loci with closely linked SNPs nearby. According to this phenomenon, Sun (Sun et al., 2020), Kureshi (Kureshi et al., 2020), and Wen (Wen et al., 2021) from our laboratory reported some greatly highly polymorphic microhaplotype loci for kinship testing, and Li (Zhao et al., 2022) from our laboratory reported some short microhaplotype loci for personal identification in forensic challenging samples. So, the highly polymorphic SNP-based microhaplotype loci may have high polymorphism and short length, which may be potential genetic markers for paternity testing and sibling testing in degraded samples. In addition, next-generation sequencing (NGS) is widely accepted by the forensic community. Illumina sequencing devices have high throughput and appropriate microhaplotype reading lengths, and NGS can directly determine the phase between SNP alleles (Bruijns et al., 2018). Therefore, NGS is considered to be the optimal strategy for microhaplotype genotyping, making short and highly polymorphic microhaplotype loci suitable for kinship analysis in degraded samples. In conclusion, this study intended to select an appropriate number of highly polymorphic SNP-based microhaplotype loci, detect them by the NGS technology, and evaluate their effectiveness in paternity testing and sibling testing, which were also compared to 15 STR loci.

## 2 Methods and materials

### 2.1 Sample collection and DNA extraction

A total of 96 whole blood samples were collected from unrelated Shandong Han Chinese. The collected samples were extracted using the universal Genomic DNA kit (CWBIO, China). The extracted DNA was quantified using NanoDrop™ one (Thermo Scientific, America). The 96 blood samples were named Sample1 to 96, and Sample8 was extracted twice to create a duplicate sample (Sample8-duplicate). The extracted DNA of Sample11 was incubated with 100°C hot water for 40 and 60 min, resulting in two degraded samples (Sample11-40, and Sample11-60). Two degraded samples were also genotyped using the AmpFLSTR^®^ Identifiler^®^ Plus PCR amplification kit (Applied Biosystems, America) and the AGCU Expressmarker 16CS PCR amplification kit (AGCU ScienTech Incorporation, China). Both kits contain the same 15 autosomal STR loci, but the AGCU Expressmarker 16CS PCR amplification kit has smaller amplicons, which is suitable for the detection of degraded samples. In addition, 2,504 individuals from 26 different populations were included in this study based on the data of the 1000 Genomes Project (Sudmant et al., 2015) (Supplementary Table S1). Written informed consent from each participant was obtained, and ethical approval was received from the Ethics Committee of Central South University (2018-S194).

### 2.2 Candidate loci

The candidate microhaplotype loci were screened based on the data of CHB of the 1000 Genomes Project (Sudmant et al., 2015) according to the following criteria: 1) Each microhaplotype locus contained two or more SNPs; 2) The allelic frequencies of SNPs within the same microhaplotype locus were different; 3) The length of each microhaplotype locus was smaller than 100 bp; 4) The *A*_*e*_ of each microhaplotype locus was larger than 3.00; 5) The heterozygosity of each microhaplotype locus was greater than 0.65; 6) The genetic distance between adjacent microhaplotype loci within the same chromosome was larger than 5 Mb. All candidate microhaplotype loci were named according to the criteria proposed by Kidd (Kidd, 2016). The details of candidate microhaplotype loci are shown in Table 1. There was a total of 36 microhaplotype loci, of which 22 loci were from Li’s study (Zhao et al., 2022), four loci from Kureshi’s study (Kureshi et al., 2020), and two loci from Wen’s study (Wen et al., 2021). Moreover, to meet the selected criteria for this study, some reported loci had SNPs deleted or SNPs added to form novel SNP combinations, which were added lower-case letters (a, b, c, … ) to the names for distinguishing them from the original combination. The other eight microhaplotype loci (loci mh01zha018, mh02zha025, mh07zha018, mh07zha027, mh10zha010, mh12zha012, mh13zha008 and mh14zha010) were firstly reported in this study.

**TABLE 1:** The details of the selected microhaplotype loci.

Locus	Chr	Position (GRCh37)	SNPs ID	Extent in bp
mh01zha018	1	4,573,068/4,573,134	**rs4568797**/rs4323680	67
mh01zha034	1	228,494,357/228,494,382	rs3795795/**rs1150911**	26
mh02zha025	2	68,237,549/68,237,578/68,237,608	rs11689307/rs11678194/**rs57008743**	60
mh02zha033	2	140,986,567/140,986,573	**rs6739332**/rs901523	7
mh03zha016	3	32,037,852/32,037,880	**rs976188**/rs17028710	29
mh04zha007	4	115,480,309/115,480,344/115,480,387	**rs6819048**/rs62308082/rs74383997	79
mh04zha012	4	18,798,844/18,798,877	**rs6820437**/rs77394386	34
mh04zha020	4	59,942,563/59,942,599	**rs11941494**/rs140524865	37
mh04zha027a	4	137,729,311/137,729,331/137,729,375	rs11936713/rs1551708/**rs1551707**	65
mh04zha031	4	166,705,962/166,705,990	**rs11935733**/rs7694605	29
mh04zha032a	4	188,290,891/188,290,908/188,290,915/188,290,948	rs3860700/**rs3860701**/rs11132442/rs3860702	58
mh05zha004a	5	174,968,649/174,968,732	rs2644662/**rs2662178**	84
mh06zha012a	6	170,554,249/170,554,250/170,554,285	rs6456186/rs6456187/**rs6456188**	37
mh06zha025	6	67,847,591/67,847,632	**rs4583967**/rs2503971	42
mh06zha026	6	77,669,385/77,669,395	rs323232/**rs323233**	11
mh07zha014	7	5,156,472/5,156,492	**rs4400288**/rs117753326	21
mh07zha018	7	11,777,712/11,777,747/11,777,769	rs9691520/**rs1534234**/rs1526523	58
mh07zha026	7	103,539,419/103,539,452/103,539,478	rs17157141/rs73183735/**rs3857817**	60
mh07zha027	7	122,708,807/122,708,851	**rs4731077**/rs4288316	45
mh08zha007a	8	4,045,817/4,045,914	**rs6996226**/rs35364155	98
mh09zha012	9	28,320,624/28,320,672	rs72709379/**rs1331923**	49
mh09zha017	9	112,618,165/112,618,187	rs10816899/**rs2769142**	23
mh09zha021	9	134,212,403/134,212,428	**rs726171**/rs2077981	26
mh10zha010	10	15,035,571/15,035,631	rs9732205/**rs9731518**	61
mh10zha020	10	126,297,208/126,297,257/126,297,261	rs11245314/rs7079225/**rs7079227**	54
mh11zha006b	11	124,823,941/124,823,950/124,823,981	rs3809057/**rs3809056**/rs3809055	41
mh11zha010	11	3,479,453/3,479,464/3,479,478	rs10834159/**rs28508343**/rs12365855	26
mh12zha012	12	713,677/713,712/713,741	**rs61916660**/rs2535397/rs11063832	65
mh12zha014	12	20,553,325/20,553,347	**rs11045217**/rs201378364	23
mh13zha003	13	59,822,210/59,822,229/59,822,240	rs2874768/**rs12870119**/rs138891898	31
mh13zha008	13	98,640,318/98,640,386	**rs592246**/rs588144	69
mh14zha003	14	72,252,135/72,252,139/72,252,142	rs4902946/rs8012670/**rs4902947**	8
mh14zha008	14	22,095,528/22,095,573	rs185847116/**rs34904279**	46
mh14zha010	14	77,688,982/77,689,033/77,689,054	rs10400728/rs72728998/**rs9323637**	73
mh16zha013	16	58,900,991/58,901,029/58,901,035	rs9935162/rs76244992/**rs9935173**	45
mh18zha010a	18	844,106/844,110/844,130/844,136/844,145	**rs7228601**/rs7236768/rs8084713/rs190860816/rs2846762	40

The multi-allelic SNPs, are marked in bold.

### 2.3 MiSeq sequencing

The multiplex amplified PCR primers for the selected loci were designed by Thermo Fisher Scientific Life Technologies. The extracted 99 DNA samples, including 96 unrelated samples and one duplicate sample and two degraded samples, were subjected to two rounds of PCR amplification (multiplex amplified PCR and index PCR) to complete the library construction, and then the constructed library was sequenced by the Illumina MiSeq sequencing platform. Reads containing adapter contamination and low-quality reads were removed from the raw data using bcl2fastq software and BBMap (version 37.75)’s BBDuk software. The clean reads were compared to the human genome (GRCh37.p13) using BWA software, and the sequencing results were analyzed using Freebayes software (Li and Durbin, 2009; Garrison and Marth, 2012). The Integrative Genomics Viewer **(**IGV) software was used to view the sequencing results (Thorvaldsdóttir et al., 2013). The values of GQ > = 20 and GQ > = 30 for each sample were greater than 0.99 and 0.90, respectively. Because a slight imbalance was observed between these loci, the sequencing reads were filtered by two different researchers to call the genotype for each locus.

### 2.4 Statistical analysis

The Log 10 values of total reads for each sample were analyzed by the histogram, and the Log 10 values of mean reads for each locus were also analyzed by the histogram. The reproducibility was analyzed by comparing the sequencing results between Sample8 and Sample8-duplicate, and the ability to detect degraded samples was evaluated by comparing the sequencing results between Sample11 and Sample11–40 and Sample11–60. The Hardy–Weinberg equilibrium was analyzed based on the exact test using a Markov chain (Guo and Thompson, 1992), and linkage equilibrium in genotypic data was analyzed based on the permutation test using the EM algorithm (Slatkin and Excoffier, 1996), which were all performed using the Arlequin version 3.5 software (Excoffier and Lischer, 2010). The forensic parameters, including allelic frequency, power of discrimination (PD), probability of exclusion (PE), and observed heterozygosity (Ho) were calculated using the modified Powerstats version 1.2 software (Zhao et al., 2003). *A*_*e*_ was also calculated according to the formula reported by Kidd (Kidd and Speed, 2015).

The 100,000 parent-child vs. 100,000 unrelated individual pairs, and 100,000 full-sibling vs. 100,000 unrelated individual pairs, and 100,000 half-sibling vs. 100,000 unrelated individual pairs, and 100,000 full-sibling vs. 100,000 half-sibling pairs were simulated using Families 3 software based on data of 15 STR loci, 29 microhaplotype loci and 79 microhaplotype loci, respectively (Kling et al., 2014). The allelic frequencies of 15 STR loci were from Luo’s study (Luo et al., 2020), the allelic frequencies of 29 microhaplotype loci were from our studied population, and the allelic frequencies of 79 microhaplotype loci were from CHB. The mutation rate of 10^–3^ and extended stepwise mutation model was applied for STR. The mutation rate of 10^–8^ and equal probability mutation model was applied for microhaplotype. The likelihood ratio (LR) values of the above four kinds of relationships were recorded as paternity index (PI), full-sibling index (FSI), half-sibling index (HSI) and full/half-sibling index (FHSI), separately. LR involves two alternative hypotheses (Hp and Hd), where Hp represents a true relationship (parent-child, full-sibling, or half-sibling) and Hd represents unrelated individuals. But for FHSI, Hp represents full-sibling and Hd represents half-sibling. The distributions of Log 10 of PI, FSI, HSI, and FHSI were analyzed, and the uncovered rates (UCR) were also calculated for these four kinds of relationship pairs. The UCR was calculated as the following formula: The number of simulated true relationship Hp (Hd) pairs larger (smaller) than the maximum (minimum) LR of simulated true relationship Hd (Hp) pairs/Total simulated true relationship Hp (Hd) pairs. The system power based on the data of 15 STR loci, 29 microhaplotype loci and 79 microhaplotype loci for the above four kinds of relationships simulated pairs at different threshold values (t_1_, t_2_) was also calculated, including sensitivity, specificity, positive predictive value (PPV), negative predictive value (NPV), error rate and effectiveness. When the Log 10 LR was larger than t_1_, the relationship Hp was supported, but when the Log 10 LR was smaller than t_2_, the relationship Hd was supported. The relationship was uncertain when the Log10 LR was between t_1_ and t_2_. The sensitivity was calculated by the formula: Number of relatives correctly judging as relatives/Number of relatives; the specificity was calculated by the formula: Number of non-relatives correctly judging as non-relatives/Number of non-relatives; the PPV was calculated by the formula: Number of relatives correctly judging as relatives/Number of judging as relatives; the NPV was calculated by the formula: Number of non-relatives correctly judging as non-relatives/Number of judging as non-relatives; the error rate was calculated by the formula: (Number of relatives judging as non-relatives + Number of non-relatives judging as relatives)/(Total relatives + Total non-relatives); the effectiveness was calculated by the formula: (Number of relatives correctly judging as relatives + Number of non-relatives correctly judging as non-relatives)/(Total relatives + Total non-relatives).

## 3 Results

### 3.1 The general information

The 36 microhaplotype loci were successfully sequenced in 96 unrelated samples (Sample1–96), one duplicate sample (Sample8-duplicate) and two degraded samples (Sample 11–40 and Sample11–60). These microhaplotype loci were located on 16 different chromosomes. One microhaplotype locus included five SNPs, one microhaplotype locus included four SNPs, 14 microhaplotype loci included three SNPs, and the other 20 microhaplotype loci included two SNPs. The length of these microhaplotype loci ranged from 7 to 98 bp, and the mean length was 45.19 bp. The example sequencing raw data of locus mh10zha010 for Sample8 is shown in Supplementary Figure S1, according to which it was genotyped as AC/GA. The Log 10 values of total reads for each sample are shown in Supplementary Figure S2, which were larger than 4.00. The Log 10 values for mean reads for each locus are presented in Supplementary Figure S3, and except for locus mh04zha020, the Log 10 values of other loci were greater than 2.00. The detailed genotyping profiles of 99 samples are listed in Supplementary Table S2. The genotyping profile of Sample8 was consistent with the duplicate sample (Sample8-duplicate), which indicated good reproducibility of our panel. The genotyping profiles of Sample11–40 and Sample11–60 were identical to that of Sample11, which suggested these microhaplotype loci had a good ability to detect degraded samples. However, when these two degraded samples were examined using the AmpFLSTR^®^ Identifiler^®^ Plus PCR amplification kit, the allele dropout was observed, and the dropout number gradually increased with increasing incubation time (Supplementary Figure S4). Even after using the AGCU Expressmarker 16CS PCR amplification kit with smaller amplicons, the allele dropout was also observed in Sample11-60 (Supplementary Figure S5). So, our microhaplotype panel may be more suitable for the detection of degraded samples than universal STR genetic markers.

### 3.2 The forensic parameters analysis

#### 3.2.1 The forensic parameters based on the data of our studied population

For total of 36 microhaplotype loci based on the data of our studied population, after the Bonferroni correction (*p* < 0.05/36 = 0.0014), seven microhaplotype loci (mh02zha025, mh02zha033, mh07zha018, mh10zha020, mh11zha010, mh12zAha012, and mh12zha014) showed significant deviations from Hardy-Weinberg equilibrium but the other 29 microhaplotype loci did not (Supplementary Table S3). The seven microhaplotype loci with significant deviations may be affected by genotyping errors (Hosking et al., 2004; Attia et al., 2010), but for the other 29 microhaplotype loci, the signals for disequilibrium may also be undetected due to the conservativeness of Bonferroni correction (Ye et al., 2020; Graffelman and Weir, 2022). The above 29 microhaplotype loci also did not observe the significant linkage disequilibrium after the Bonferroni correction (*p* < 0.05/406 = 0.0001), which is presented in Supplementary Table S4. So, only 29 microhaplotype loci were included in the subsequent analysis.

The forensic parameters of 29 microhaplotype loci based on the data of our studied population are listed in Table 2. A total of 140 alleles were observed, and the locus mh04zha032a had the largest number of 13 alleles. The smallest PD value was obtained in the locus mh05zha004a (0.83), and the largest PD value was obtained in the locus mh04zha032a (0.91). The PE values had the range of 0.27 (mh14zha010) to 0.58 (mh06zha025). The combined power of discrimination (CPD) for these 29 microhaplotype loci was 1-2.96E-26, while the combined probability of exclusion (CPE) was 1-5.45E-09. The Ho values ranged from 0.58 (mh14zha010) to 0.79 (mh06zha025), and the mean Ho was 0.73. The mean *A*_*e*_ was 3.61, and the *A*_*e*_ values of 29 microhaplotype loci were all larger than 3.00. These results indicated that our microhaplotype panel had a good potential for personal identification and kinship testing.

**TABLE 2:** Allelic frequencies and forensic parameters of the 29 microhaplotype loci based on the dataset of 96 unrelated Shandong Han Chinese from our study.

**mh01zha018**		**mh01zha034**		**mh03zha016**		**mh04zha007**		**mh04zha012**		**mh04zha020**		**mh04zha027a**		**mh04zha031**		**mh04zha032a**		**mh05zha004a**	
TC	0.24	TC	0.14	AT	0.17	AAT	0.40	AT	0.36	TC	0.24	AGT	0.11	TA	0.16	TAAA	0.05	TC	0.22
CC	0.34	CT	0.33	TC	0.01	AAC	0.18	AC	0.23	CT	0.41	CAT	0.21	TG	0.33	TAAG	0.01	GA	0.35
GT	0.11	CC	0.35	CT	0.32	CAC	0.17	TC	0.19	CC	0.18	CGT	0.19	CG	0.34	TTCG	0.01	GC	0.08
GC	0.30	CG	0.18	CC	0.21	GGC	0.26	CC	0.22	GT	0.01	CGC	0.39	GG	0.17	TGAA	0.02	GG	0.35
				GT	0.29					GC	0.17	CGG	0.10			TGAG	0.14		
																TGCA	0.02		
																TGCG	0.06		
																CAAA	0.47		
																CAAG	0.09		
																CACG	0.02		
																CTCG	0.03		
																CGAG	0.06		
																CGCG	0.05		
																			
PD	0.86	PD	0.87	PD	0.88	PD	0.87	PD	0.86	PD	0.85	PD	0.89	PD	0.84	PD	0.91	PD	0.83
PE	0.53	PE	0.46	PE	0.44	PE	0.43	PE	0.56	PE	0.55	PE	0.58	PE	0.56	PE	0.46	PE	0.46
Ho	0.76	Ho	0.72	Ho	0.71	Ho	0.70	Ho	0.78	Ho	0.77	Ho	0.79	Ho	0.78	Ho	0.72	Ho	0.72
*A*_*e*_	3.57	*A*_*e*_	3.54	*A*_*e*_	3.81	*A*_*e*_	3.54	*A*_*e*_	3.74	*A*_*e*_	3.53	*A*_*e*_	3.94	*A*_*e*_	3.58	*A*_*e*_	3.84	*A*_*e*_	3.32
**mh06zha012a**		**mh06zha025**		**mh06zha026**		**mh07zha014**		**mh07zha026**		**mh07zha027**		**mh08zha007a**		**mh09zha012**		**mh09zha017**		**mh09zha021**	
ATA	0.35	AC	0.37	AC	0.17	AT	0.32	TTC	0.22	TC	0.33	AA	0.34	AT	0.23	TT	0.25	AA	0.23
ATC	0.30	TC	0.23	GA	0.33	TT	0.23	TGA	0.23	CT	0.32	TA	0.29	GT	0.22	TC	0.17	AG	0.33
ATG	0.02	GT	0.10	GC	0.36	CT	0.31	TGT	0.38	CC	0.21	TC	0.24	GC	0.22	TG	0.39	TG	0.17
GAG	0.15	GC	0.30	GG	0.14	CC	0.14	TGC	0.10	GC	0.14	CA	0.13	GG	0.33	CG	0.19	GG	0.28
GTG	0.18							CGA	0.07										
																			
PD	0.89	PD	0.83	PD	0.86	PD	0.87	PD	0.87	PD	0.87	PD	0.88	PD	0.89	PD	0.87	PD	0.88
PE	0.32	PE	0.58	PE	0.49	PE	0.47	PE	0.55	PE	0.46	PE	0.43	PE	0.38	PE	0.47	PE	0.56
Ho	0.63	Ho	0.79	Ho	0.74	Ho	0.73	Ho	0.77	Ho	0.72	Ho	0.70	Ho	0.67	Ho	0.73	Ho	0.78
*A*_*e*_	3.73	*A*_*e*_	3.47	*A*_*e*_	3.47	*A*_*e*_	3.70	*A*_*e*_	3.82	*A*_*e*_	3.65	*A*_*e*_	3.62	*A*_*e*_	3.87	*A*_*e*_	3.57	*A*_*e*_	3.79
**mh10zha010**		**mh11zha006b**		**mh13zha003**		**mh13zha008**		**mh14zha003**		**mh14zha008**		**mh14zha010**		**mh16zha013**		**mh18zha010a**	
AA	0.30	AAT	0.26	TTT	0.04	TA	0.14	CAT	0.10	TA	0.05	TGC	0.48	TTT	0.39	ATGGA	0.28		
AC	0.43	ATC	0.33	TTC	0.01	TG	0.19	CCA	0.36	TG	0.10	GTG	0.12	TTC	0.15	CCAGG	0.09		
AG	0.18	AGC	0.20	TCT	0.11	CA	0.36	CCT	0.05	GA	0.26	GGA	0.10	TTG	0.07	CCGGG	0.39		
GA	0.09	GGC	0.21	TCC	0.02	GG	0.31	GCA	0.21	GC	0.11	GGC	0.05	CTC	0.24	GTAAG	0.13		
				TGT	0.18			GCG	0.29	GG	0.48	GGG	0.24	CCC	0.15	GTAGG	0.01		
				GTT	0.05											GTGGA	0.11		
				GCT	0.14														
				GGT	0.46														
																			
PD	0.85	PD	0.88	PD	0.88	PD	0.84	PD	0.89	PD	0.83	PD	0.85	PD	0.88	PD	0.88		
PE	0.39	PE	0.49	PE	0.46	PE	0.49	PE	0.38	PE	0.49	PE	0.27	PE	0.56	PE	0.51		
Ho	0.68	Ho	0.74	Ho	0.72	Ho	0.74	Ho	0.67	Ho	0.74	Ho	0.58	Ho	0.78	Ho	0.75		
*A*_*e*_	3.19	*A*_*e*_	3.83	*A*_*e*_	3.53	*A*_*e*_	3.52	*A*_*e*_	3.75	*A*_*e*_	3.08	*A*_*e*_	3.11	*A*_*e*_	3.86	*A*_*e*_	3.74		

#### 3.2.2 The forensic parameters based on the data of the 1000 Genomes Project

**FIGURE 1:**
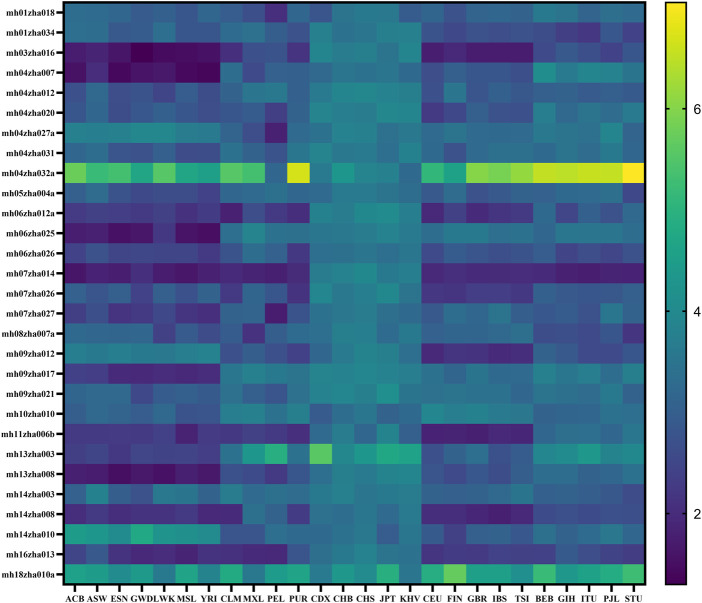
The heatmap of *A*_*e*_ distribution of 29 microhaplotype loci in 26 populations based on the data of the 1000 Genomes Project.

The heatmap of *A*_*e*_ distribution of 29 microhaplotype loci in 26 populations based on the data of the 1000 Genomes Project is shown in Figure 1. The loci mh04zha032a and mh18zha010a were highly polymorphic in all 26 populations with *A*_*e*_ larger than 3.00. The populations on the same continent had similar *A*_*e*_ distributions, for example, ACB, ASW, ESN, GWD, LWK, MSL, and YRI in AFR; CLM, MXL, PEL and PUR in AMR; CDX, CHB, CHS, JPT, and KHV in EAS; CEU, FIN, GBR, IBS, and TSI in EUR; BEB, GIH, ITU, PJL, and STU in SAS. But the populations CLM and PUR of AMR were more similar to the *A*_*e*_ distribution of EUR. The polymorphism of EAS was higher than that of the other four continents, and the polymorphism of AFR was the worst. The CPD values of 29 microhaplotype loci in 26 populations ranged from 1-1.60E-19 (YRI) to 1-4.89E-27 (CHS), and the CPE values ranged from 1-2.62E-05 (YRI) to 1-2.28E-09 (CHS). These results suggested our microhaplotype panel was more polymorphic in EAS and can discriminated between different populations.

### 3.3 The pairwise kinship testing

#### 3.3.1 The pairwise kinship testing based on 15 STR loci

**FIGURE 2:**
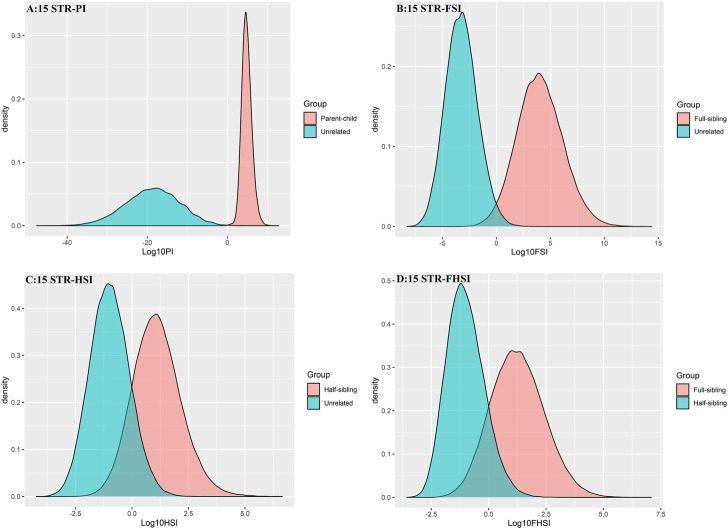
The distributions of Log 10 of PI, FSI, HSI, and FHSI based on the data of 15 STR loci. **(A)** PI; **(B)** FSI; **(C)** HSI; **(D)** FHSI.

The distributions of Log 10 of PI, FSI, HSI and FHSI based on the data of 15 STR loci are presented in Figure 2. For relationship parent-child vs. unrelated individuals, a slight overlap was observed, and some degree of overlap was obtained in relationship full-sibling vs. unrelated individuals. There was a significant overlap in relationship half-sibling vs. unrelated individuals and full-sibling vs. half-sibling. The UCR values for these four kinds of relationship pairs based on the data of 15 STR loci are shown in Table 3. The UCR for relationship parent-child vs. unrelated individuals was 86.70% of true relationship Hp and 98.51% of true relationship Hd. The UCR was smaller than 60% for relationship full-sibling vs. unrelated individuals, and smaller than 10% for relationship half-sibling vs. unrelated individuals and full-sibling vs. half-sibling. The system power based on the data of 15 STR loci for relationship parent-child vs. unrelated individuals at different threshold values is listed in Figure 5. When the threshold was set as 4/−4 for relationship parent-child vs. unrelated individuals, the sensitivity, specificity, error rate and effectiveness were 74.50%, 98.99%, 0.00%, and 86.75%, separately. The sensitivity, specificity, error rate and effectiveness were 83.51%, 79.95%, 0.07%, and 81.73% at the threshold of 2/−2 for relationship full-sibling vs. unrelated individuals (Figure 6). But for relationship half-sibling vs. unrelated individuals and full-sibling vs. half-sibling (Figure 7 and Figure 8), the effectiveness values were about 50% and the error rates reached 1% even at the threshold of 1/−1. These 15 STR loci were only suitable for paternity testing, but the effectiveness of paternity testing at the threshold of 4/−4 was also smaller than 90%.

**TABLE 3:** The UCR values of four kinds of relationship pairs using the 15 STR loci, 29 microhaplotype loci and 79 microhaplotype loci.

Dataset	Relationship	Max (Hd|Log10LR)	UCR(Hp|Log10LR > Max (Hd|Log10LR))	Min (Hp|Log10LR)	UCR(Hd|Log10LR < Min (Hp|Log10LR))
15 STR	Parent-child vs. Unrelated	3.51	86.70%	−4.79	98.51%
	Full-sibling vs .Unrelated	3.84	52.70%	−4.89	12.79%
	Half-sibling vs. Unrelated	3.24	3.01%	−2.87	1.16%
	Full-sibling vs. Half-sibling	3.46	3.43%	−2.47	2.15%
29 MH	Parent-child vs. Unrelated	2.81	99.75%	−3.74	99.99%
	Full-sibling vs. Unrelated	4.60	62.04%	−4.72	53.38%
	Half-sibling vs. Unrelated	3.77	1.10%	−3.49	1.96%
	Full-sibling vs. Half-sibling	3.64	8.58%	−3.25	5.52%
79 MH	Parent-child vs. Unrelated	−57.62	100.00%	5.26	100.00%
	Full-sibling vs. Unrelated	1.13	99.99%	−0.37	100.00%
	Half-sibling vs. Unrelated	3.75	48.32%	−4.14	38.97%
	Full-sibling vs. Half-sibling	3.74	71.52%	−4.34	51.34%

#### 3.3.2 The pairwise kinship testing based on 29 microhaplotype loci

**FIGURE 3:**
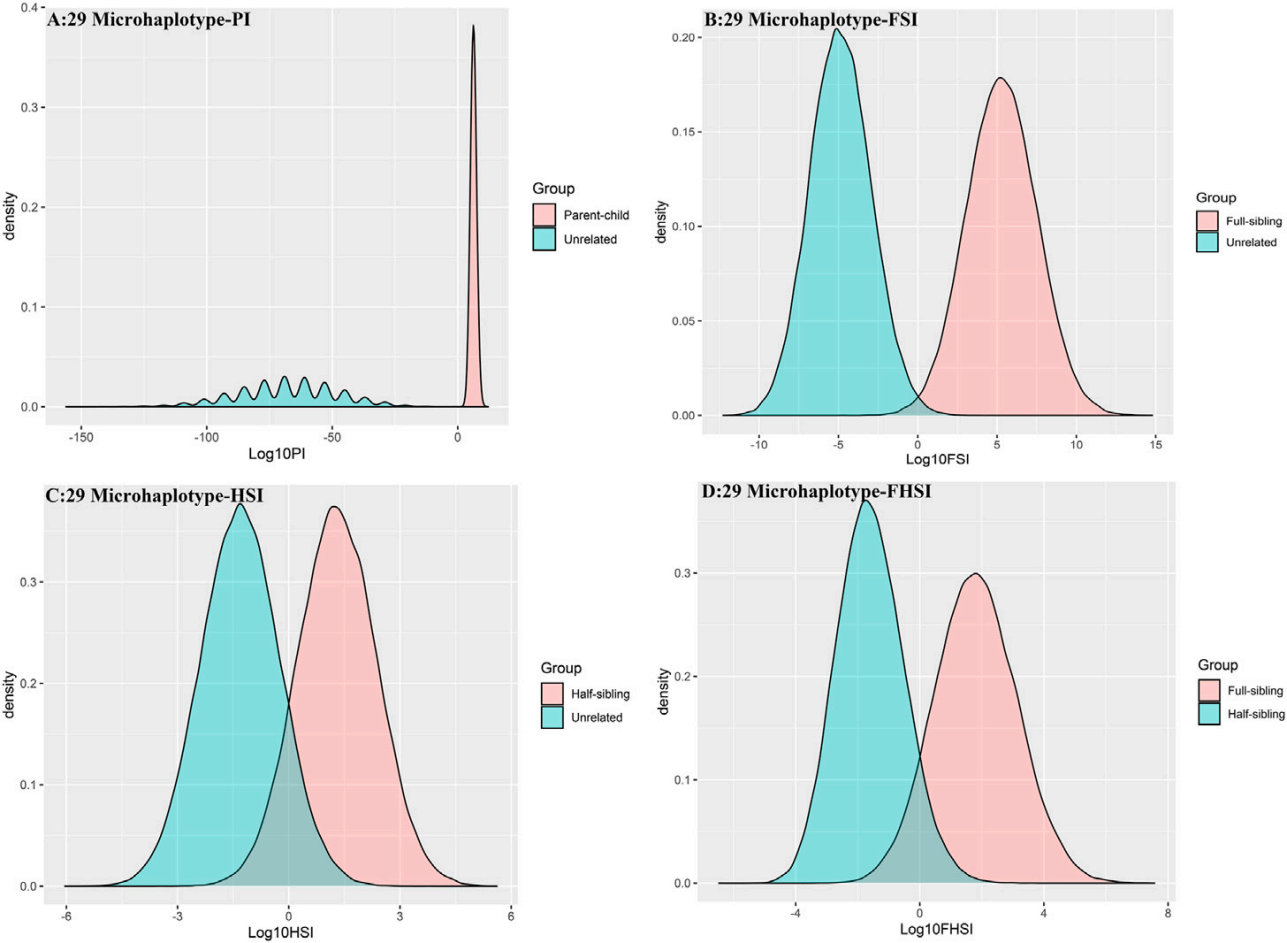
The distributions of Log 10 of PI, FSI, HSI and FHSI based on the data of 29 microhaplotype loci. **(A)** PI; **(B)** FSI; **(C)** HSI; **(D)** FHSI.

The distributions of Log 10 of PI, FSI, HSI and FHSI based on the data of 29 microhaplotype loci are presented in Figure 3. After using the 29 microhaplotype loci, the mean Log 10 LR values for four kinds of relationship pairs with true relationship Hp were larger than that of 15 STR loci, and mean Log 10 LR values for four kinds of relationship pairs with true relationship Hd were smaller than that of 15 STR loci, especially for unrelative pairs in relationship parent-child vs. unrelated individuals due to the lower mutation rate. For relationship parent-child vs. unrelated individuals, no overlap was observed, and a slight overlap was obtained in relationship full-sibling vs. unrelated individuals, which were greatly smaller than that of 15 STR loci. There was also a significant overlap in relationship half-sibling vs. unrelated individuals and full-sibling vs. half-sibling. The UCR values for these four kinds of relationship pairs based on the data of 29 microhaplotype loci are shown in Table 3. The UCR for relationship parent-child vs. unrelated individuals was larger than 99%. The UCR was about 60% for relationship full-sibling vs. unrelated individuals. The UCR values were also smaller than 10% for relationship half-sibling vs. unrelated individuals and full-sibling vs. half-sibling. The system power based on the data of 29 microhaplotype loci for relationship parent-child vs. unrelated individuals at different threshold values is listed in Figure 5. When the threshold was set as 4/−4 for relationship parent-child vs. unrelated individuals, the sensitivity, specificity, error rate and effectiveness were 96.79%, 99.99%, 0.00% and 98.39%, separately. The sensitivity, specificity, error rate and effectiveness were 93.30%, 92.72%, 0.03%, and 93.01% at the threshold of 2/−2 for relationship full-sibling vs. unrelated individuals (Figure 6). But for relationship half-sibling vs. unrelated individuals and full-sibling vs. half-sibling (Figure 7 and Figure 8), the effectiveness values were 61.33% and 71.98% even at the threshold of 1/−1. These results suggested the system power of 29 microhaplotype loci was greater than that of 15 STR loci, and our microhaplotype panel had a good ability in paternity testing and full sibling testing. But for the identification of more distant kinship relationship, our system still needs to be supplemented by other loci.

#### 3.3.3 The pairwise kinship testing based on 79 microhaplotype loci

**FIGURE 4:**
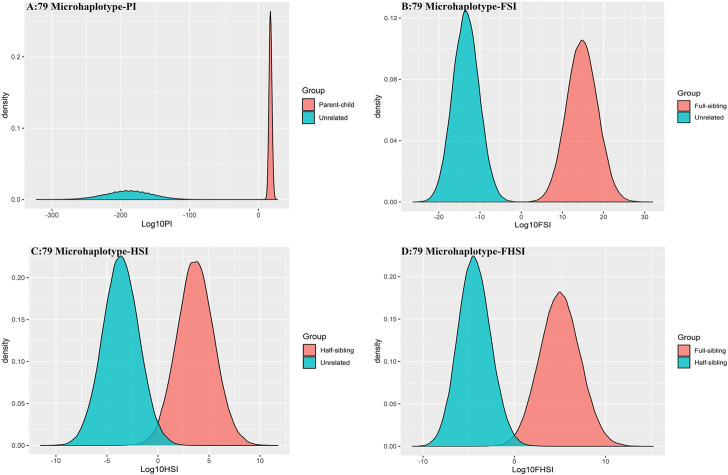
The distributions of Log 10 of PI, FSI, HSI and FHSI based on the data of 79 microhaplotype loci. **(A)** PI; **(B)** FSI; **(C)** HSI; **(D)** FHSI.

**FIGURE 5:**
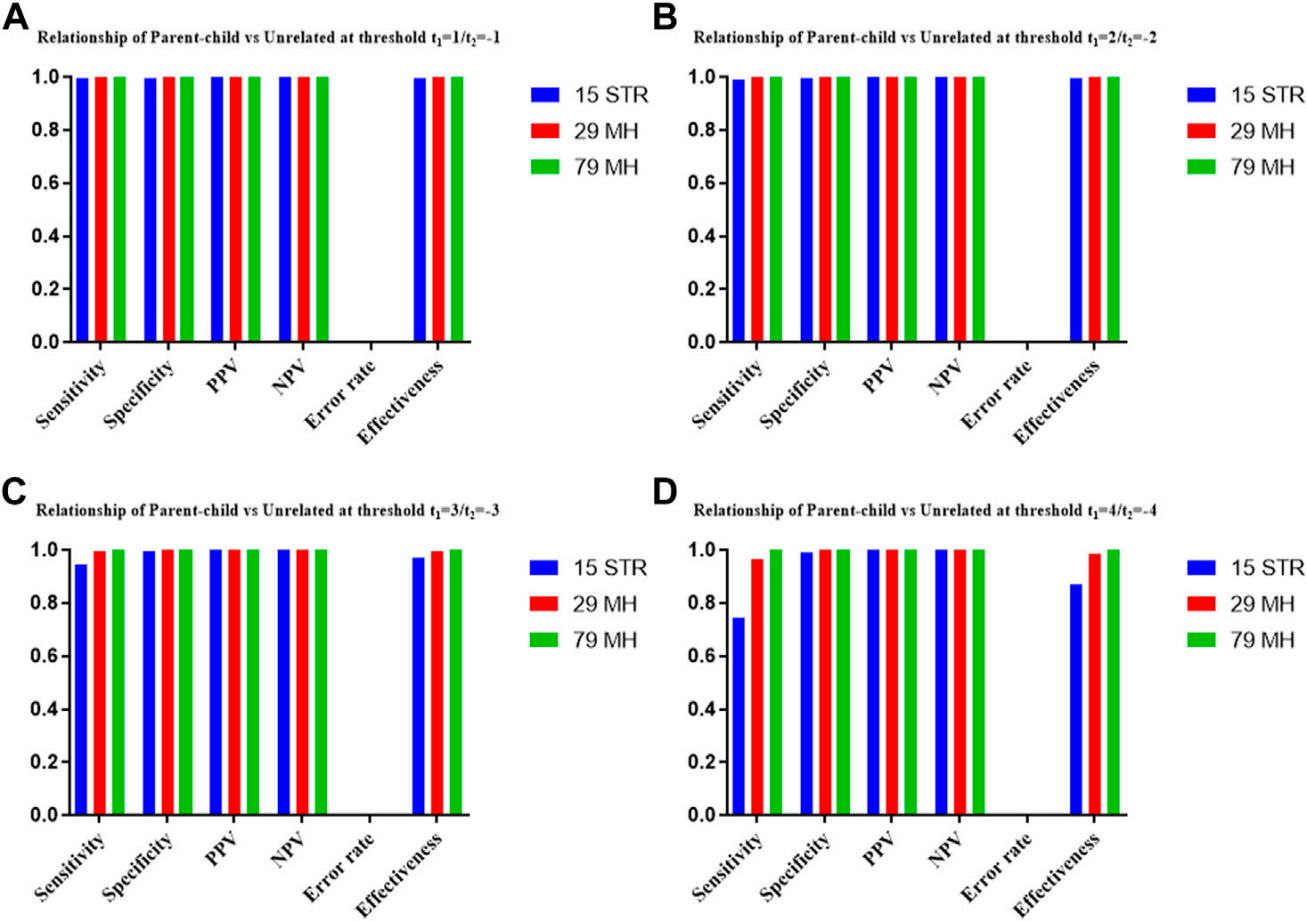
The system power based on the data of 15 STR loci, 29 microhaplotype loci and 79 microhaplotype loci for relationship parent-child vs. unrelated individuals at different threshold values. **(A)** Threshold t_1_ = 1/t_2_ = −1; **(B)** Threshold t_1_ = 2/t_2_ = −2; **(C)** Threshold t_1_ = 3/t_2_ = −3; **(D)** Threshold t_1_ = 4/t_2_ = −4.

**FIGURE 6:**
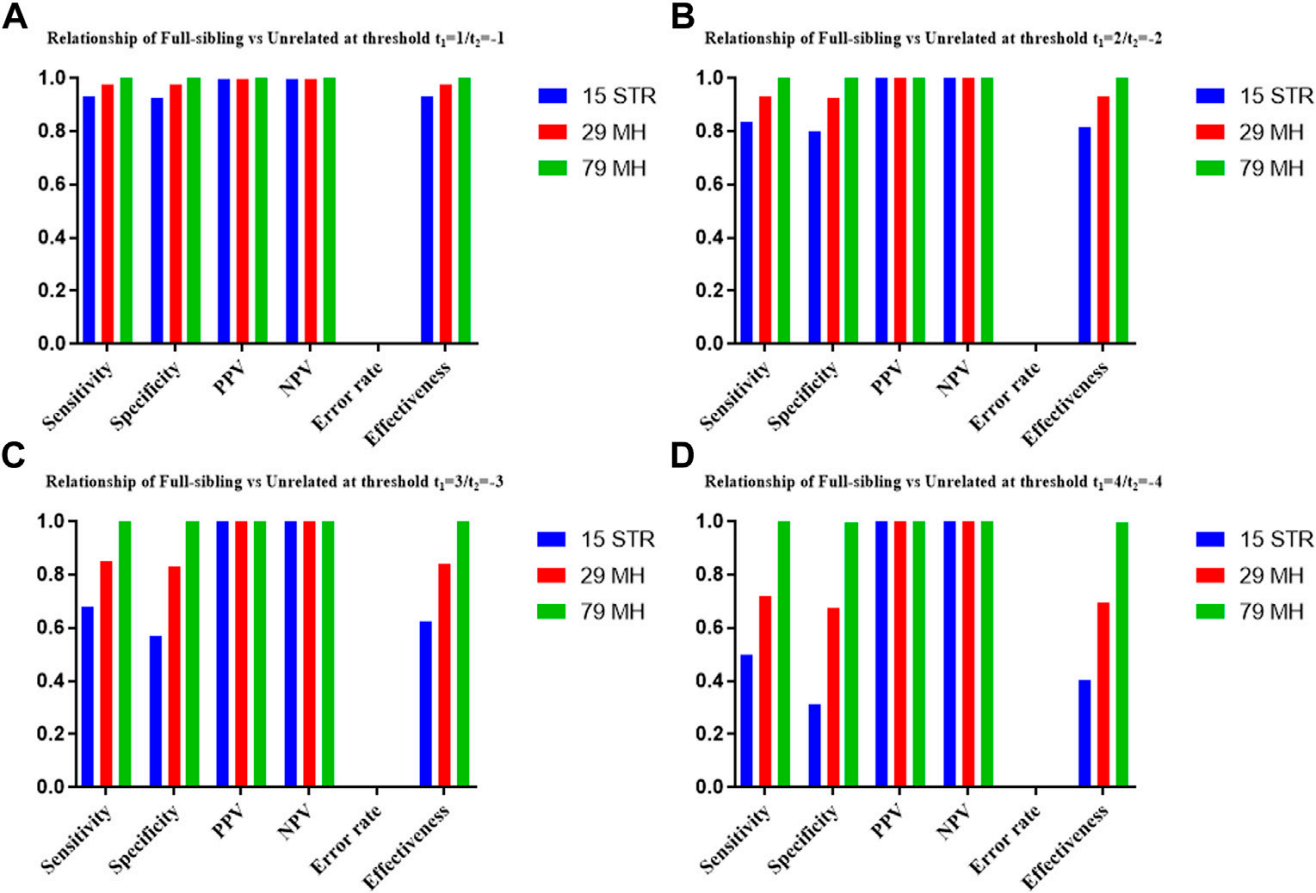
The system power based on the data of 15 STR loci, 29 microhaplotype loci and 79 microhaplotype loci for relationship full-sibling vs. unrelated individuals at different threshold values. **(A)** Threshold t_1_ = 1/t_2_ = −1; **(B)** Threshold t_1_ = 2/t_2_ = −2; **(C)** Threshold t_1_ = 3/t_2_ = −3; **(D)** Threshold t_1_ = 4/t_2_ = −4.

**FIGURE 7:**
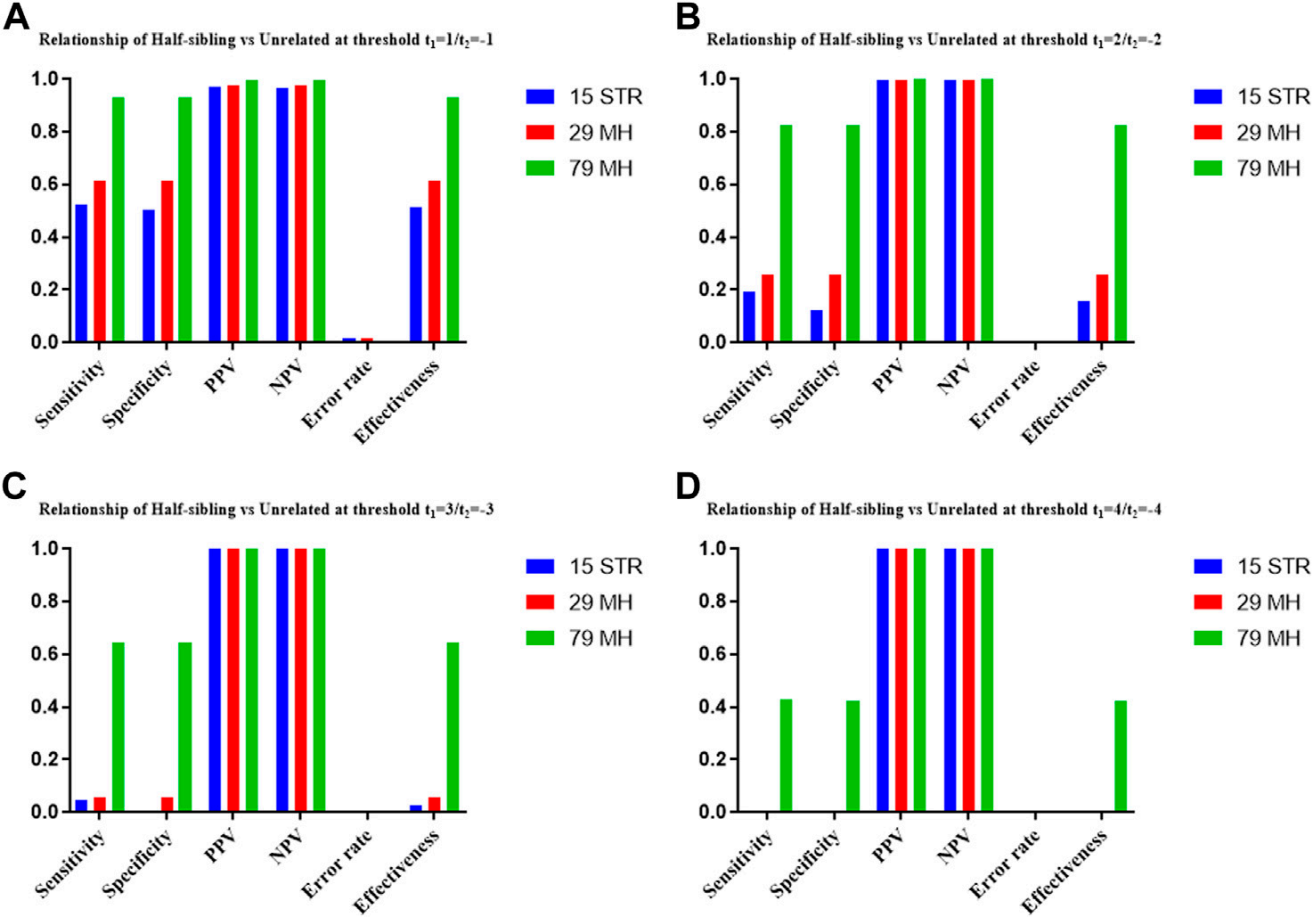
The system power based on the data of 15 STR loci, 29 microhaplotype loci and 79 microhaplotype loci for relationship half-sibling vs. unrelated individuals at different threshold values. **(A)** Threshold t_1_ = 1/t_2_ = −1; **(B)** Threshold t_1_ = 2/t_2_ = −2; **(C)** Threshold t_1_ = 3/t_2_ = −3; **(D)** Threshold t_1_ = 4/t_2_ = −4.

**FIGURE 8:**
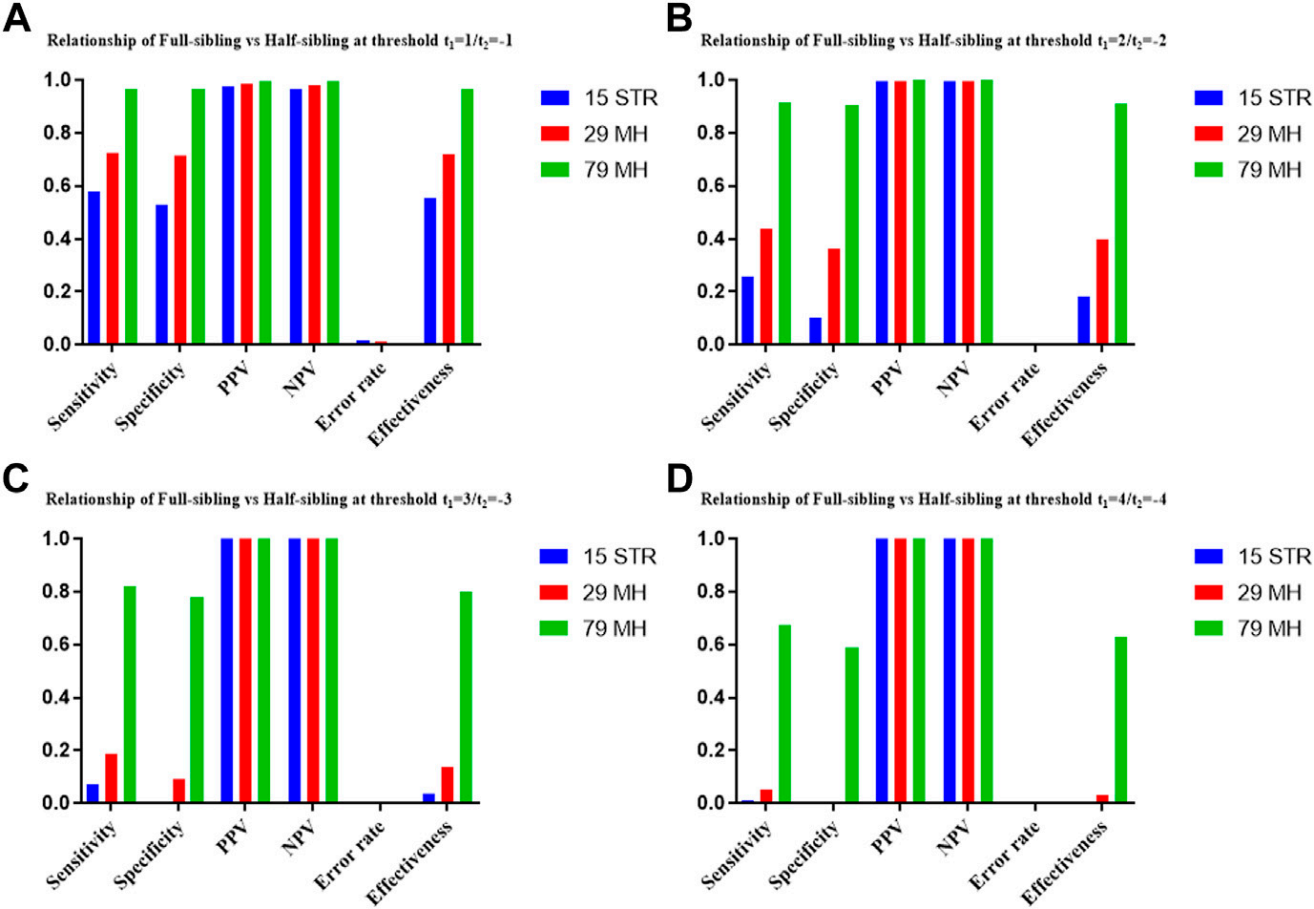
The system power based on the data of 15 STR loci, 29 microhaplotype loci and 79 microhaplotype loci for relationship full-sibling vs. half-sibling at different threshold values. **(A)** Threshold t_1_ = 1/t_2_ = −1; **(B)** Threshold t_1_ = 2/t_2_ = −2; **(C)** Threshold t_1_ = 3/t_2_ = −3; **(D)** Threshold t_1_ = 4/t_2_ = −4.

To further improve the ability of half sibling testing and to distinguish full sibling from half sibling in degraded samples, our 29 microhaplotype loci were combined with other 50 short and highly polymorphic microhaplotype loci (Supplementary Table S5). A total of 79 short and highly polymorphic microhaplotype loci were included in the simulated kinship testing, of which 29 microhaplotype loci were reported by our study, and 9 microhaplotype loci were reported by Staading’s study (Staadig and Tillmar, 2021), and 41 microhaplotype loci were reported by the Puente’s study (de la Puente et al., 2020). For simulated kinship analysis, the allelic frequencies of 79 microhaplotype loci were from CHB, and the linkage equilibrium was assumed. The distributions of Log 10 of PI, FSI, HSI and FHSI based on the data of 79 microhaplotype loci are presented in Figure 4. For relationship parent-child vs. unrelated individuals and full-sibling vs. unrelated individuals, no overlap was observed, and a slight overlap was obtained in relationship half-sibling vs. unrelated individuals and full-sibling vs. half-sibling. The UCR values for these four kinds of relationship pairs based on the data of 79 microhaplotype loci are shown in Table 3. The UCR values for relationship parent-child vs. unrelated individuals and full-sibling vs. unrelated individuals reached 100%. The UCR values were also about 50% for relationship half-sibling vs. unrelated individuals and full-sibling vs. half-sibling. The system power based on the data of 79 microhaplotype loci for relationship parent-child vs. unrelated individuals and relationship full-sibling vs. unrelated individuals at different threshold values is listed in Figure 5 and Figure 6. When the threshold was set as 4/−4 for relationship parent-child vs. unrelated individuals and full-sibling vs. unrelated individuals, the sensitivity, specificity, PPV, NPV and effectiveness values were larger than 99%, and the error rate values were 0.00%. The sensitivity, specificity, error rate and effectiveness were 82.40%, 82.44%, 0.07%, and 82.42% at the threshold of 2/-2 for relationship half-sibling vs. unrelated individuals (Figure 7). For relationship full-sibling vs. half-sibling (Figure 8), the effectiveness was about 90.89% and the error rate reached 0.05% at the threshold of 2/−2. These 79 microhaplotype loci can completely distinguish the parent-child from unrelated individuals and full-sibling from unrelated individuals, and had a strong ability to identify half-sibling and distinguish full-sibling from half-sibling. The combined short and highly polymorphic microhaplotype panel may be very useful for the complex kinship analysis in degraded samples.

## 4 Discussion

In this study, we developed a short and highly polymorphic microhaplotype panel containing 36 highly polymorphic SNP-based microhaplotype loci with the length smaller than 100 bp and *A*_*e*_ greater than 3.00, of which 29 microhaplotype loci could not reject the Hardy-Weinberg equilibrium and linkage equilibrium after the Bonferroni correction. The CPD and CPE of these 29 microhaplotype loci were 1-2.96E-26 and 1-5.45E-09, respectively, and no allele dropout was observed in degraded samples incubated with 100°C hot water for 40 and 60 min. The developed microhaplotype panel may be suitable for personal identification and kinship testing in degraded samples. According to the simulated kinship analysis, the effectiveness at the threshold of 4/−4 reached 98.39% for relationship parent-child vs. unrelated individuals, and the effectiveness at the threshold of 2/−2 for relationship full-sibling vs. unrelated individuals was 93.01%, which was greater than that of 15 STR loci (86.75% for relationship parent-child vs. unrelated individuals and 81.73% for relationship full-sibling vs. unrelated individuals). After combining our 29 microhaplotype loci with 50 short and highly polymorphic microhaplotype loci reported by Staading’s study and Puente’s study, the effectiveness values were 82.42% and 90.89% at the threshold of 2/−2 for relationship half-sibling vs. unrelated individuals and full-sibling vs. half-sibling. Our developed short and highly polymorphic microhaplotype panel may be very useful for paternity testing and full sibling testing in degraded samples, and in combination with short and highly polymorphic microhaplotype loci reported by other researchers, may be helpful to analyze more distant kinship relationships.

Although the 15 autosomal STR loci included in the AmpFlSTRTM IdentifilerTM Plus PCR Amplification Kit and the AGCU Expressmarker 16CS PCR amplification kit were still the main loci used in paternity testing and personal identification according to Luo’s study (Luo et al., 2020), Hill’s study also reported a better-powered combination of 29 autosomal STR loci with a mean Ho of 0.81 (Hill et al., 2013). The combination of 29 microhaplotype loci in our developed panel had better performance than the combination of 15 autosomal STR loci, but the microhaplotype loci in our panel should be combined with other short and highly polymorphic microhaplotype loci to achieve the performance of other kits containing a large number of STR loci. Our developed panel may be very useful for first-degree relationship testing in degraded samples, and when combined with other 50 short and highly polymorphic microhaplotype loci, may be helpful for second-degree relationship testing. But the third-degree relationship testing, such as first cousin testing, can also be observed in forensic cases. The effectiveness of relationship first-cousin vs. unrelated individuals was 11.48% at the threshold of 2/−2 after simulated kinship analysis using the Families 3 software based on the data of 79 microhaplotype loci. To simplify the simulation, the mutation rates of 79 microhaplotype loci were set to 0. The combination of 79 microhaplotype loci, including 29 microhaplotype loci in our developed panel and the other 50 short and highly polymorphic microhaplotype loci, had limited performance in third-degree relationship testing. To address these complex and distant kinship relationship analyses, more microhaplotype loci are needed. After analyzing the *A*_*e*_ distribution of 29 microhaplotype loci in 26 populations based on the data of the 1000 Genomes Project, it was found that five continents had different polymorphisms, and the polymorphism of EAS was higher than that of the other four continents. Therefore, the developed panel is more suitable for paternity testing and personal identification in EAS, while the construction of population-specific microhaplotype panels may also be useful for other populations. Moreover, the detection of degraded samples collected from real cases can provide deeper insight into the applicability of our panel, so we will use our panel in further studies to detect samples exposed to various degradable conditions and degraded samples collected from real cases.

## Data Availability

The original contributions presented in the study are publicly available. This data can be found here: https://www.ncbi.nlm.nih.gov/ PRJNA858268
