# Supplementary material for: The application of short and highly polymorphic microhaplotype loci in paternity testing and sibling testing of temperature-dependent degraded samples
Source: Front Genet. 2022 Sep 26;13:983811. doi: 10.3389/fgene.2022.983811 (PMC9549137; doi:10.3389/fgene.2022.983811)
Supplement: Supplementary file 3 [file Table1.DOCX]

**Supplementary Table 1.** The details of 26 populations from the 1000 Genomes Project.

| Continents | Populations |
| --- | --- |
| African(AFR) | Afro-Caribbean in Barbados (ACB) |
|  | Americans of African ancestry in south western USA (ASW) |
|  | Esan in Nigeria (ESN) |
|  | Gambian in Western Divisions in the Gambia (GWD) |
|  | Luhya in Webuye, Kenya (LWK) |
|  | Mende in Sierra Leone (MSL) |
|  | Yoruba from Ibadan, Nigeria (YRI) |
| American (AMR) | Colombians from Medellin, Colombia (CLM) |
|  | Mexican ancestry from Los Angeles, USA (MXL) |
|  | Peruvians from Lima, Peru (PEL) |
|  | Puerto Ricans from Puerto Rico (PUR) |
| East Asian (EAS) | Chinese Dai in Xishuangbanna, China (CDX) |
|  | Chinese in Beijing, China (CHB) |
|  | Southern Han Chinese (CHS) |
|  | Japanese in Tokyo Japan (JPT) |
|  | Kinh in Ho Chi Minh City, Vietnam (KHV) |
| Europeans (EUR) | Residents of Utah, USA with Northern and Western European Ancestry (CEU) |
|  | Finnish in Finland (FIN) |
|  | British in England and Scotland (GBR) |
|  | Iberian population in Spain (IBS) |
|  | Toscani in Italy (TSI) |
| South Asian (SAS) | Bengali from Bangladesh (BEB) |
|  | Gujarati Indian from Houston, Texas, USA (GIH) |
|  | Indian Telugu from the UK (ITU) |
|  | Punjabi from Lahore, Pakistan (PJL) |
|  | Sri Lankan Tamil from the UK (STU) |
