# Supplementary figures and images for: The application of short and highly polymorphic microhaplotype loci in paternity testing and sibling testing of temperature-dependent degraded samples
Source: Front Genet. 2022 Sep 26;13:983811. doi: 10.3389/fgene.2022.983811 (PMC9549137; doi:10.3389/fgene.2022.983811)

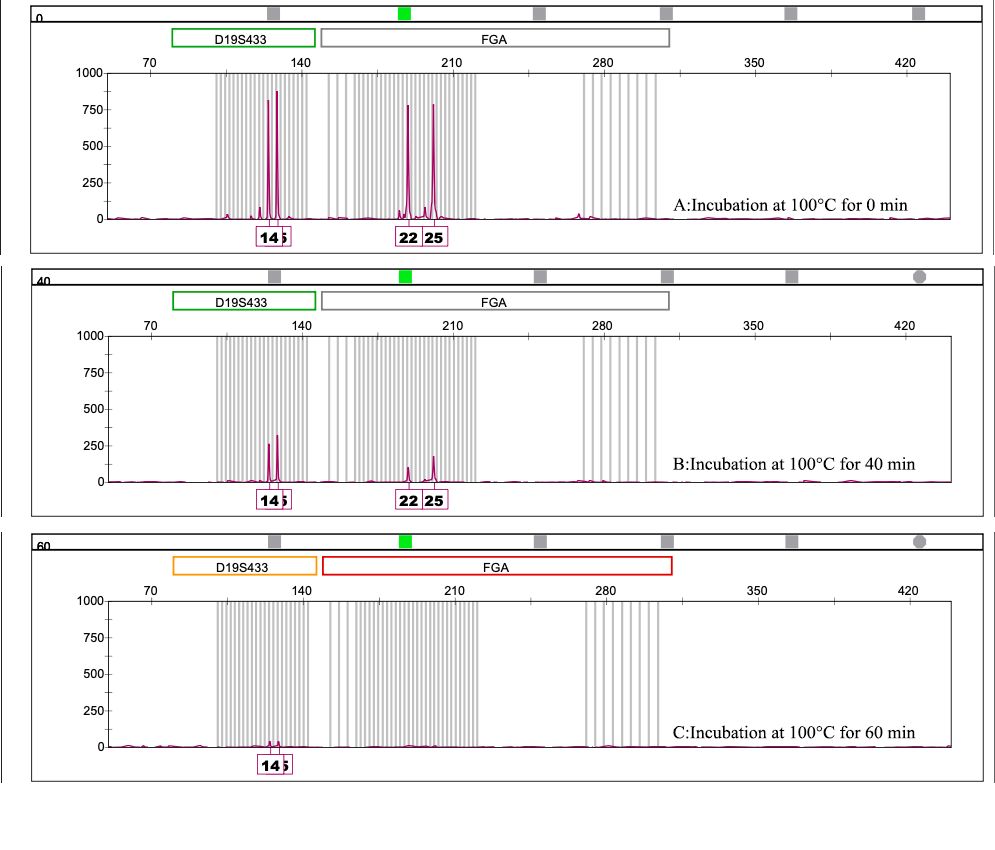

Supplement: Supplementary file 4 [file Image5.PNG]

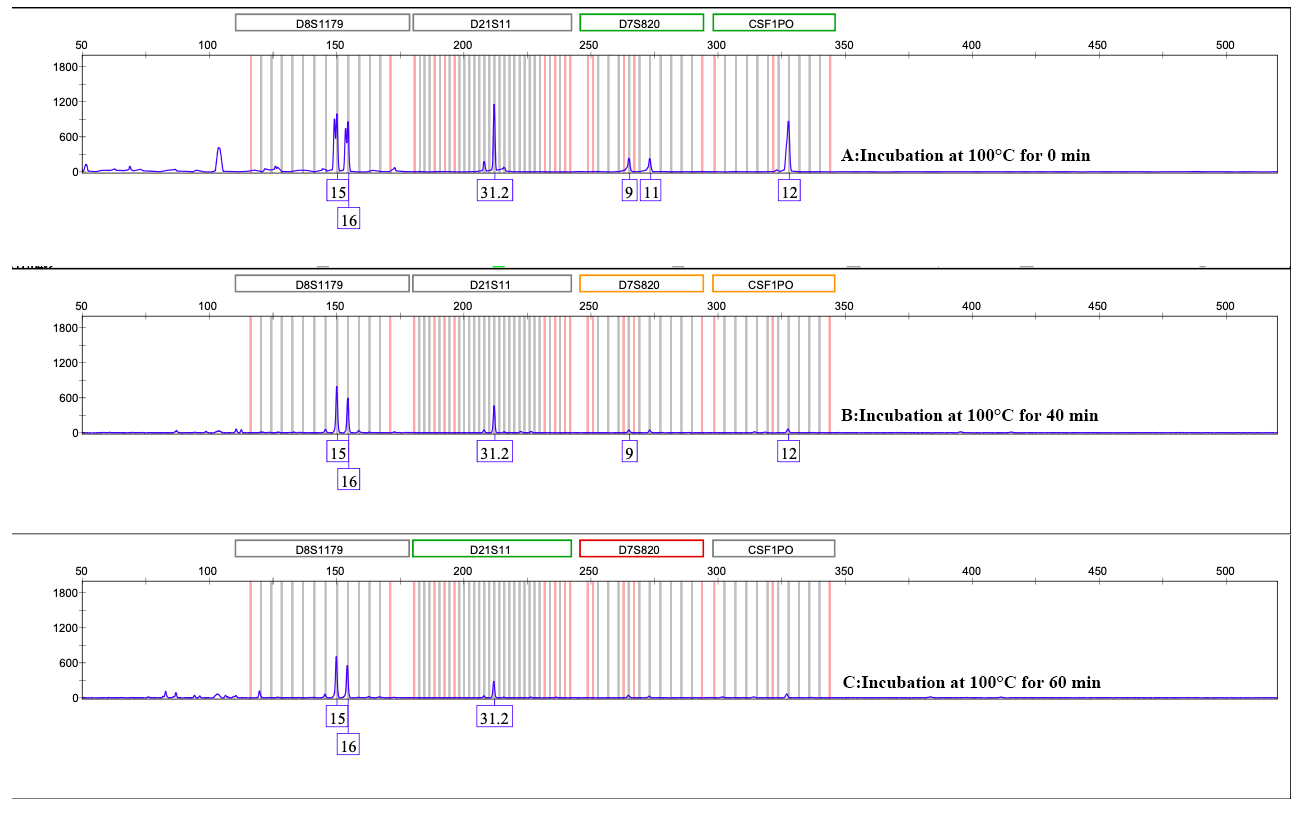

Supplement: Supplementary file 5 [file Image4.PNG]

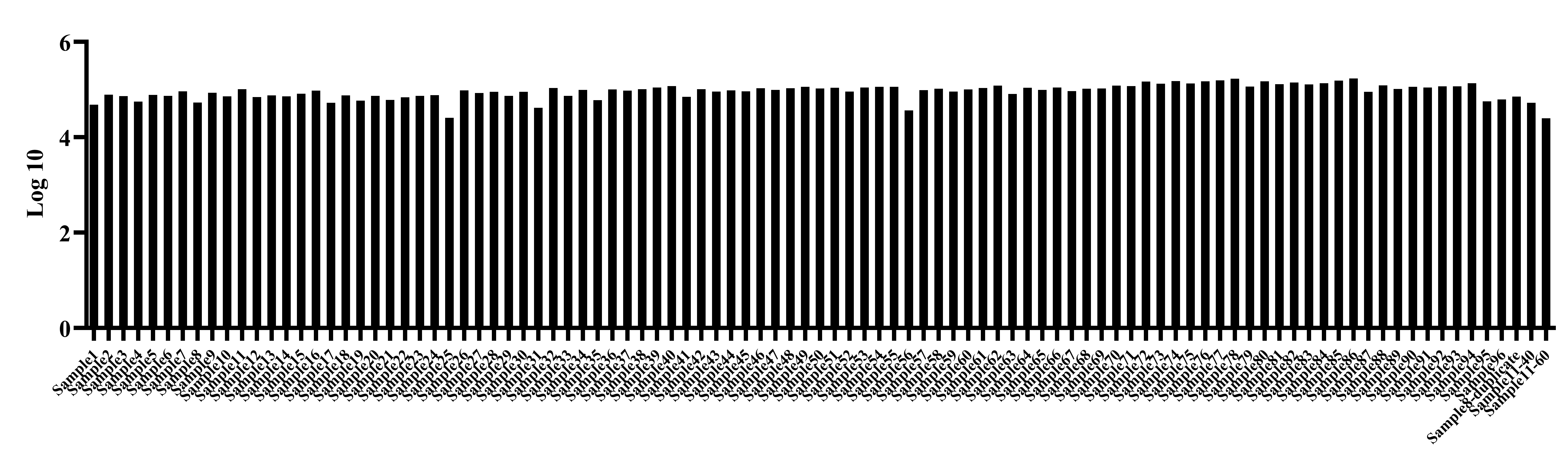

Supplement: Supplementary file 7 [file Image2.PNG]

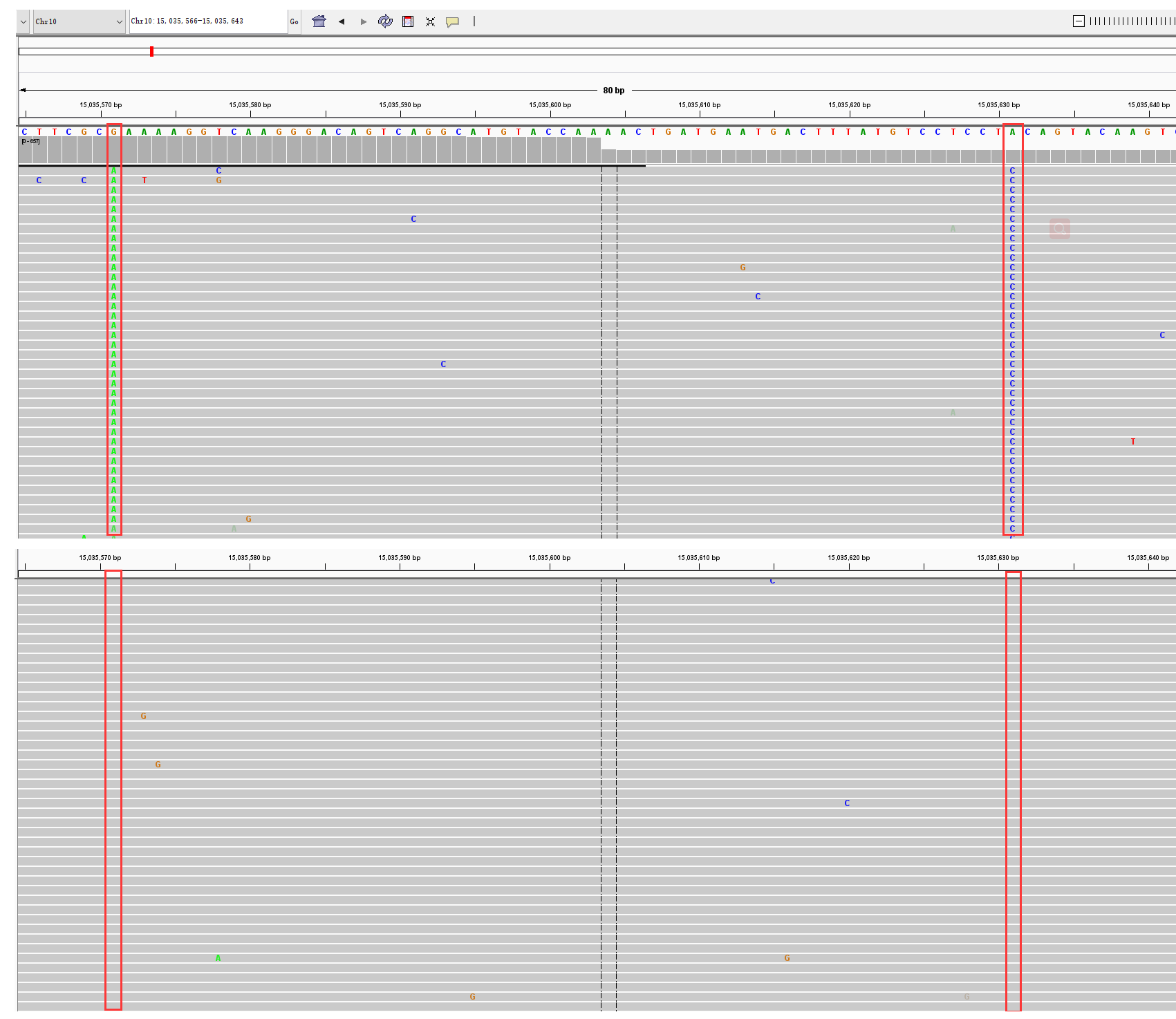

Supplement: Supplementary file 8 [file Image1.PNG]

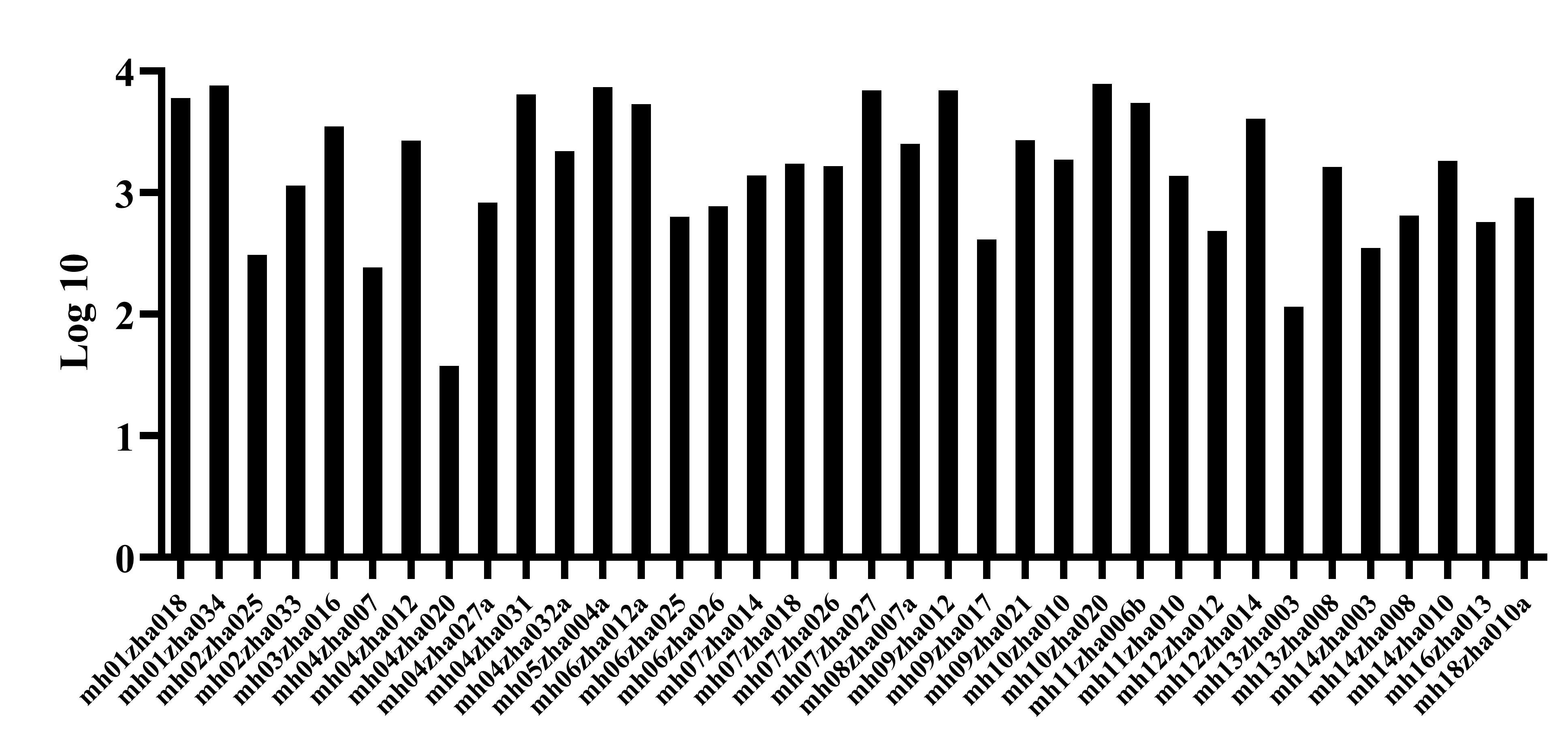

Supplement: Supplementary file 10 [file Image3.PNG]
